# The evolutionary origins and significance of drug addiction

**DOI:** 10.1186/1477-7517-2-8

**Published:** 2005-06-29

**Authors:** Tammy Saah

**Affiliations:** 1 Stanford University School of Medicine, Transplant Immunobiology Laboratory, USA

## Abstract

By looking at drug addiction from an evolutionary perspective, we may understand its underlying significance and evaluate its three-fold nature: biology, psychology, and social influences. In this investigation it is important to delve into the co-evolution of mammalian brains and ancient psychotropic plants. Gaining an understanding of the implications of ancient psychotropic substance use in altering mammalian brains will assist in assessing the causes and effects of addiction in a modern-day context. By exploring addiction in this manner, we may move towards more effective treatment early prevention, treating the root of the issue rather than the symptoms.

## 1. Introduction

As we find ourselves in the beginning of a new millennium, we are faced with challenges to our survival as a human population. Some of the greatest threats to our survival are sweeping epidemics that affect millions of individuals worldwide. Drug addiction, although often regarded as a personality disorder, may also be seen as a worldwide epidemic with evolutionary genetic, physiological, and environmental influences controlling this behavior. Globally, the use of drugs has reached all-time highs. On average, drug popularity differs from nation to nation. The United Nations Office on Drugs and Crime identified major problem drugs on each continent by analyzing treatment demand [1]. From 1998 to 2002, Asia, Europe, and Australia showed major problems with opiate addiction, South America predominantly was affected by cocaine addiction, and Africans were treated most often for the addiction to cannabis. Only in North America was drug addiction distributed relatively evenly between the use of opiates, cannabis, cocaine, amphetamines, and other narcotics. However, all types of drugs are consumed throughout each continent. Interpol reported over 4000 tons of cannabis were seized in 1999, up 20% from 1998, with the largest seizures made in Southern Africa, the US, Mexico, and Western Europe [2]. Almost 150 tons of cocaine is purchased each year throughout Europe and in 1999 opium production reached an estimated 6600 tons, the dramatic increase most likely due to a burst of poppy crops throughout Southwest Asia. This rapid increase in drug use has had tremendous global effects, and the World Health Organization cited almost 200,000 drug-induced deaths alone in the year 2000 [3]. The Lewin group for the National Institute on Drug Abuse and the National Institute on Alcohol Abuse and Alcoholism estimated the total economic cost of problematic use of alcohol and drugs in the United States to be $245.7 billion for the year 1992, of which $97.7 billion was due to drug abuse [4]. The White House Office of National Drug Control Policy (ONDCP) found that between 1988 and 1995, Americans spent $57.3 billion on drugs, of which $38 billion was on cocaine, $9.6 billion was on heroin and $7 billion was on marijuana.

Among the different approaches for diagnosis, prevention, and treatment of drug addiction, exploring the evolutionary basis of addiction would provide us with better understanding since evolution, personality, behavior and drug abuse are tightly interlinked. It is our duty as scientists to explore the evolutionary basis and origins of drug addiction so as to uncover the underlying causes rather than continuing to solely focus on the physiological signs and global activity of this epidemic. Too often the treatment of addiction simply works to alleviate the symptoms of addiction, dealing with overcoming the physiological dependence and working through withdrawal symptoms as the body readjusts to a non-dependent state of homeostasis. However, we must not only concentrate on this aspect of addiction when considering global treatments and preventative programs. We must take into consideration that it is not purely the physiology of addiction we are battling.

Drug addiction is thought of as an adjunctive behavior, or a subordinate behavior catalyzed by deeper, more significant psychological and biological stimuli. It is not just a pharmacological reaction to a chemical but a mode of compensation for a decrease in Darwinian fitness [5]. There are three main components involved in substance addiction: developmental attachment, pharmacological mechanism, and social phylogeny including social inequality, dominance, and social dependence [6]. Developmental attachment created by environmental influences, such as parental care or lack thereof, may influence children's vulnerability to drug addiction. Evolutionarily speaking, children that receive care that is more erratic may focus more so on short-term risks that may have proved to be an adaptive quality for survival in ancient environments. Compounding that attachment, the pharmacological mechanism describes the concept of biological adaptation of the mesolimbic dopamine system to endogenous substance intake. These factors combined with the influence of social phylogeny create a position for predisposition to drug addiction. They attribute to the common belief that many substances of abuse have great powers to heal, and that is often the driving motivation for overuse and addiction. Evolutionary perspective shows an intermediate and fleeting expected gain associated with drug addiction correlated with the conservation in most mammals of archaic neural circuitry [7], most often being a falsified sense of increased fitness and viability related to the three components of drug abuse [5,8]. The chemical changes associated with fitness and viability are perceived by mammals as emotions, driving human behavior.

Human behavior is mediated primarily by dopaminergic and serotonergic systems, both of ancient origins probably evolving before the phylogenetic splits of vertebrates and invertebrates [9]. 5-HT (serotonin), stimulated by a small range of drugs, mediates arousal. It is believed to be inhibited by hallucinogens and also helps control wanting for ethanol and cocaine consumption. The cortico-mesolimbic dopaminergic system, on the other hand, is believed to be the target of a wide range of drugs, including marijuana and cocaine, increasing the transmission of dopamine to the nucleus accumbens [10]. This system mediates emotion and controls reinforcement, and is the primary pathway acted on by antipsychotic drugs such as chlorprothixene and thioridazine. Problematic use of drugs develops into addiction as the brain becomes dependent on the chemical neural homeostatic circuitry altered by the drug [7]. No matter the theory of drug addiction, there remains one constant: withdrawal is inevitable. As a drug is administered continuously and an individual becomes addicted, the brain becomes dependent on the presence of the drug. With an absence of the drug, withdrawal symptoms are experienced as the brain attempts to deal with the chemical changes. There are believed to be evolutionary origins of drug addiction, which will be discussed further, as well as a link between physiological addiction and the evolution of emotion.

## 2. Drugs distribution and use in ancient environments

When examining the distribution of natural drugs in ancestral environment we see that there was often a limited amount of resources, meaning there was little overactivity of salient (wanting) behavior, causing no need for the adaptive development within the cortico-mesolimbic dopaminergic system of a built-in regulatory system of salience [6,11]. Genetic and environmental factors increasing substance abuse liability may have been of no consequence in ancestral environments due to their limitations. We originally relied on the limitations of ancient environments in that same manner, so when we are introduced to excessive amounts of salience in modern environment, we have no internal control. Basically, our ancient-wired bodies have not yet evolved to adapt to modern environment, leaving us vulnerable to addiction.

A common belief is that psychotropic plant chemicals evolved recurrently throughout evolutionary history [12]. Archaeological records indicate the presence of psychotropic plants and drug use in ancient civilizations as far back as early hominid species about 200 million years ago. Roughly 13,000 years ago, the inhabitants of Timor commonly used betel nut (*Areca catechu*), as did those in Thailand around 10,700 years ago. At the beginning of European colonialism, and perhaps for 40,000 years before that, Australian aborigines used nicotine from two different indigenous sources: pituri plant (*Duboisia hopwoodii*) and *Nicotiana gossel*. North and South Americans also used nicotine from their indigenous plants *N. tabacum *and *N. rustica*. Ethiopians and northern Africans were documented as having used an ephedrine-analog, khat (*Catha edulis*), before European colonization. Cocaine (*Erythroxylum coca*) was taken by Ecuadorians about 5,000 years ago and by the indigenous people of the western Andes almost 7,000 years ago. The substances were popularly administered through the buccal cavity within the cheek. Nicotine, cocaine, and ephedrine sources were first mixed with an alkali substance, most often wood or lime ash, creating a free base to facilitate diffusion of the drug into the blood stream. Alkali paraphernalia have been found throughout these regions and documented within the archaeological record. Although the buccal method is believed to be most standard method of drug administration, inhabitants of the Americas may have also administered substances nasally, rectally, and by smoking.

Many indigenous civilizations displayed a view of psychotropic plants as food sources, not as external chemicals altering internal homeostasis [12]. The perceived effects by these groups were tolerance to thermal fluctuations, increased energy, and decreased fatigue, all advantageous to fitness by allowing longer foraging session as well as greater ability to sustain in times of limited resources. The plants were used as nutritional sources providing vitamins, minerals, and proteins rather than recreational psychotropic substances inducing inebriation. Due to limited resources within ancient environments, mammalian species most probably sought out CNS neurotransmitter (NT) substitutes in the form of psychotropic allelochemicals, because nutrient NT-precursors were not largely available in the forms of food. Therefore, drugs became food sources to prevent decreased fitness from starvation and death. It is believed that early hominid species evolved in conjunction with the psychotropic flora due to constant exposure with one another. This may be what eventually allowed the above civilizations to use the flora as nutritional substances, therefore increasing both their fitness and viability.

Over time, psychotropic plants evolved to emit allelochemical reactivity to deter threats from herbivores and pathogenic invasions. These allelochemical responses evolved to imitate mammalian NT so as to act as competitive binders and obstruct normal CNS functioning. The allelochemical NT analogs were not anciently as potent as forms of abused substances used in modern environments, but instead were milder precursors that had an impact on the development of the mammalian CNS. The fit of allelochemicals within the CNS indicates some co-evolutionary activity between mammalian brains and psychotropic plants, meaning they interacted ecologically and therefore responded to one another evolutionarily. Basically, series of changes occurred between the mammalian brain and psychotropic plants allowing them affect one another during their processes of evolving. This would have only been possible with mammalian CNS exposure to these allelochemicals, therefore to ancient mammalian psychotropic substance use. The evidence for this theory is compelling. For example, the mammalian brain has evolved receptor systems for plant substances, such as the opioid receptor system, not available by the mammalian body itself. The mammalian body has also evolved to develop defenses against overtoxicity, such as exogenous substance metabolism and vomiting reflexes.

## 3. Evolutionary advantage of emotion

The evolution of brain systems brought about indicators of levels of fitness in the form of chemical signals perceived as emotion [7,8,11,13]. These emotions help direct physiology and behavior of an individual towards increasing Darwinian fitness. They essentially were tools chosen for by the mechanisms of natural selection. Positive emotions, such as euphoria and excitation, motivate towards increased gain and fitness state, whereas negative emotions, for instance anxiety and pain, evolved as defenses by motivating towards managing potential threats or decreases in fitness level.

Mammalian drive to escape danger is fueled by a capacity to feel negative emotions [14]. Negative emotions can be defenses, and in their suppression we may find ourselves unarmed and unprepared to deal with problems much more detrimental than the original warning emotions. Those individuals that lack the capacity to suffer, including the inability to experience pain, are unable to put up basic physiological and behavioral defenses and often find themselves dying at relatively young ages. Negative emotions (pain, fear, stress, anxiety, etc.) have evolved in mammals to allude to even the slightest, most harmless potential indicator of a more serious problem, leading to what may be known as a modern-day personality disorder. Personality disorders can be characterized as anything from over-anxiety to schizophrenia [13]. Many emotional disorders that drugs mask, such as anxiety disorders, develop from the ancient adaptive mechanisms expressed by the evolved mode of personality, and may in fact not be disorders but hypersensitive neural adaptations. Since personality evolved as an information gating mechanism to transmit culture among people, as well as within an individual from external environmental stimuli to internal neural circuitry for personal regulation, negative emotion may be simply transmitted and can be enhanced through personality [15].

There are two defined types of positive emotion [7]. The first includes feelings of anticipation and excitation induced by a promise of an increase in fitness (+ Positive Affect, or PA), while the second includes emotions of relief and security due to a removal of a threat to fitness (- Negative Affect or NA). + PA emotions fall into the behavioral activation system, or the BAS [16]. The BAS attempts to propagate positive emotions and appetitive conditioning, resulting in a motivation to reach goals and, essentially, the positive affect. – NA emotions fall into the behavioral inhibition system (BIS), which attempts to regulate and compensate for negative emotions and aversive conditioning. As mammals expose themselves to fitness-increasing situations and avoid fitness-decreasing situations, they tend to motivate towards pleasure-inducing, or + PA, stimuli that indicate these increases in fitness. Even if unrelated to fitness in modern environment, emotions continue to process events in the same archaic way. Many pleasant feelings may now not indicate an increase in fitness at all, but the evolutionary brain may still correlate the two.

Modern environments include medical and social technologies that bring comfort and longer living than was experienced in ancient environment, so much of modern human emotion does not serve the same function as was evolutionarily performed. As our emotions become less indicative of fitness and more superfluous, there comes to be confusion within the intended signals of emotion. The pursuit of "happiness" involves gain, and while evolutionarily these gains were increased fitness, the emotion of happiness is no longer directly related to fitness. While one may become happy due to a casual and pleasing relationship, the euphoric emotion may have evolutionarily corresponded with an indication of successful reproduction and therefore a gain in fitness and viability. This can also be applied to the euphoria associated with wealth, which in ancient environments may have been an indicator of increases in fitness due to plentiful food and water resources, but now may indicate status.

## 4. Effects of drugs on emotion

Psychoactive drugs induce emotions that at one point in mammalian evolutionary history signaled increased fitness, not happiness [11]. In ancient environments positive emotion correlated with a sign of increased fitness, such as successful foraging sessions or successful breeding. Mammals would feel euphoric only during times where fitness levels were high, the euphoria being indicative of survival and not a superfluous feeling of "happiness." Mammals would otherwise feel negative emotions when fitness levels were low. The effect of many psychoactive substances provided the same euphoric feeling, and may have had some increasing effects on fitness levels in ancient mammalian species. However, drug use today does not carry the same predicted increases in fitness, and in fact may act as a pathogen on neural circuitry. Yet, these same drugs continue to target archaic mechanisms of the brain with the intent of inducing positive emotion, essentially blocking many neurological defenses.

Drugs that stimulate positive emotion virtually mediate incentive motivation in the nucleus accumbens and the neural reward system [11]. Modern drug addiction fundamentally indicates a false increase of fitness, leading to increasing drug abuse to continue gain, even if the gain is realized as being false. This is the quintessential paradox among drug addicts. The motivation towards gain begins to take precedence over adaptive behaviors among addicted individuals. Some stimuli that simulate increased fitness may become greater priorities than true adaptive stimuli necessary for increased fitness, such as food and sleep [7]. Individuals can, in turn, decrease their fitness by ignoring necessary behaviors for survival and fitness and focusing on a false positive emotion. The appetite for a drug may also override the drive to consummate, causing a drastic decrease in viability. Their emotional systems are now concentrated on drug-seeking rather than survival.

In modern humans, drugs that may block negative emotions may be more useful than the endurance of ancient warnings of harm, like pain and fever [11]. Certain drugs can aid in pathology treatment, and while negative emotions may have been entirely necessary for the survival of ancient mammals, they may no longer be exclusively indicative of nociceptive or otherwise harmful stimuli [11,13]. Hypersensitivity of our bodies' defense mechanisms has evolved, leading to unnecessary negative emotions for non-nociceptive stimuli as preventative defense. When there is a threat towards an individual's fitness, the modern body often responds with several different warning signs, perhaps several different types of negative emotions (pain, fever, and hallucination, for example). Therefore, blocking a few of the negative emotions will ideally not disrupt the message. I emphasize the word "ideally" for this is not always the case. Frequently there are situations in which drugs that block these defenses, such as anxiolytics, may contribute to the decreases in fitness by temporarily removing a small negative emotions but leaving the individual vulnerable to a much larger harm [17].

Emotional disposition has shown to specifically correlate with problematic use of alcohol [16]. If the perceived emotion before alcohol consumption is negative, the individual most likely is drinking to cope, with less control over his/her own use. In the case of a positive disposition before consumption, the user is said to drink to enhance, with more greatly controlled use of the substance. Since alcohol consumption alters normally functioning cognitive processes, it does not prove to be equal to evolutionarily superior internal coping mechanisms. Instead, alcohol mediates not only negative feelings by their suppression, but also encourages the habituated continuance of positive emotion. Recovering alcoholics often document reasons of relapse surrounding the drive to compensate for negative feelings, resulting in a motivation to cope and therefore to drink.

## 5. Physiology of addiction and reward

Mammalian brains work heavily on a motivational system with two types of motivation: like and want [11]. Like is controlled by opioid and brain stem systems, and refers to pleasure upon receiving a reward, whereas want (salience), mediated by the cortico-mesolimbic dopaminergic system, is an anticipatory motivation to pursue reward. We receive "pleasure" through intracellular signaling of adaptive chemical pathways of a reward system that bring our attention to what we need. The nucleus accumbens (NAcb) and globus pallidus are involved in reward pathways for alcohol, opiates, and cocaine [18]. NTs involved in these pathways are dopamine (primarily within the NAcb and hippocampus), serotonin (hypothalamus), enkephalins (ventral tegmental area and NAcb), GABA (inhibitory – ventral tegmental area and NAcb), and norepinephrine (hippocampus). When there is a disturbance within the reward intracellular cascade, a chemical imbalance occurs that triggers negative emotions to be indicative of the disturbance. This is referred to as "reward deficiency syndrome," where the chemical imbalances within the intracellular cascade manifest themselves as behavioral disorders, indicating a deficiency within the adaptive reward pathway. Drug addiction may initially cause and then further proceed to exacerbate "reward deficiency syndrome."

Another theory of drug addiction, the "drugs for reward" theory, states that addiction is the malfunctioning collision of both motivational systems (like vs. want), stimulating pursuit of a substance that most probably no longer provides pleasure and in fact may be pathogenic [11]. Different drugs stimulate different types of positive emotion [7]. Opioids contribute to – NA states, while dopamine-releasing drugs contributes to + PA states. In this theory, dopamine is believed to mediate a state of addiction through the activation of the cortico-mesolimbic system passing through the ventral tegmental area to the nucleus accumbens, all regulating reward-seeking motivation. It is also involved in withdrawal from psychostimulants, as the sudden removal of a chemical drug stimulant from the body causes a massive alteration within the dopaminergic system, leading to negative emotions. Opioids are believed to mediate the consumption of reward, with opioid addiction following a well-defined route: 1) first ensues as a pleasure-seeking behavior, 2) tolerance to the opioid builds and pleasure resulting from drug use reduces, yet use is increased in an attempt to regain the hedonic pleasure, and 3) withdrawal may occur with a cessation of the opioid substance differing from withdrawal from psychostimulants, but also leading to negative emotions. With the "drugs for reward" theory, adaptive hard-wired (physiologically determined to serve a specific role) dopamine function is believed to induce a feeling of reward for a particular action that indicates an increase in the level of fitness of an individual [6]. It encourages the continuation of habit that increases dopamine release, therefore leading to a perception of increased levels of fitness (although often falsely when referring to drug use). Problems with this theory are encountered when we take into consideration that dopamine also signals negative reinforcement, not just positive reinforcement through reward. Dopamine is therefore referred to as simply altering an emotional state from one to another, even if it means going from positive emotion to negative emotion.

Dopamine is otherwise argued to be a mediator of salience [6]. Although dopamine is believed to control the cortico-mesolimbic system, it does not rule the consummatory/satiatory/seeking behavior in this particular theory. It instead mediates appetitive/approach behavior, placing an importance on things by demanding attention on either their strength (positive emotion) or their potential harm (negative emotion), then increasing the motivation to move towards an action to change, not to satiate (stop). If upregulated, a feeling of "wanting" is induced for a specific substance, leading to addiction with overuse [10]. This explains dopamine action as integrated activity rather than hard-wired function, and best explains how drug addiction is obsessively saliatory without ever reaching satiation. This concept is referred to as IS, or incentive salience. Earlier theories discussed unconditioned stimuli, such as a specific drug, as stimulants of an unconditioned response of neural regulation [19]. In this model, the drug is not the unconditioned stimulus causing guaranteed changes of the CNS, as was previously thought, but the chemical activity caused by the drug within the CNS is the unconditioned stimuli. The brain then becomes adapted to the chemical response of the drug, producing a salient conditioning response within the brain's association context. The prefrontal cortex directs associative context, in turn regulating the cortico-mesolimbic dopamine system to induce an amalgamation of abnormal behavior and salience; the individual is now driven by uncontrolled craving and wanting. We originally relied on the limitations of the ancestral environment to be the regulatory influences as we used drugs for food, and our bodies still remain adapted to ancestral environments in that aspect. Therefore, when we are introduced to excessive amounts of salience, we have no internal control.

Candidate gene polymorphisms within the above pathway receptors may contribute to substance abuse [20]. Substance abuse tendencies and liabilities (the vulnerability to a disease and the possibility of becoming affected due to genetic and environmental susceptibility) may be inherited through phenotypic liabilities. The expression of substance abuse is therefore dependent on this phenotypic liability and environmental influences. The phenotypic liability may be a result of a genetic polymorphism within the DRD2 dopamine receptor gene (A.sub.1 allele) [18]. The DRD2 dopamine receptors are targeted by antipsychotics [9]. This particular receptor gene polymorphism correlates with alcohol and substance addiction as well as obsessive compulsive disorders. The DRD4 dopamine receptor has documented polymorphisms within a 48 base pair variable number tandem repeat, and also correlates with substance addiction, for it is believed to be involved in reducing sensitivity to methamphetamines, alcohol, and cocaine. In Israeli and Arab heroin-dependent populations, there was data collected displaying a DRD4 gene polymorphism in exon 3 consisting of seven-repeat alleles not present in non-addicted control groups. This was also observed in a study of heroin-addicted Han Chinese. In a study done with Native American alcoholics, a linkage on chromosome 11 near the DRD4 gene was documented. With these phenotypic liabilities, an individual may be considered to be addicted to a substance after passing a threshold of which there is no diagnostic or solid definition. Dependence is often continued because of temporary positive effects with the denial of the more permanent, negative pharmaceutical effects. There have been documented significant relationships between drug and alcohol dependence and certain genetic factors, with the same genetic correlation to smoking, displaying a significant cohesion between different substance use disorders. Individuals addicted to substances may, therefore, be genetically predisposed to the situation and are then pushed past threshold by environmental stimuli.

## 6. Social-cultural impact

We have discovered that the nature of addiction is not solely based on free will to use, or an individual's conscious choice to use, but may have deeper influences. The nature of drug addiction is three-fold: biological, psychological, and social. Although humans may be biologically and psychologically predisposed to drug use and addiction, they may often be driven towards that state by social and cultural influences. To what extent environmental stimuli affect a person's vulnerability to addiction is unknown and may be varying. However, we cannot ignore the great impact of environmental and mental stimuli in the progression towards addiction. It has been found that certain environmental variables breed higher vulnerability [21]. Family dysfunction and disruption, low social class rearing, poor parental monitoring, and rampant social drug-use exposure may greatly contribute to an individual's movement from substance abuse predisposition to addiction. Both acute and chronic stresses have been linked with substance abuse as well, with acute stress being one of the main influences of relapse in rehabilitated drug addicts. The widespread availability of drugs in certain areas also may affect susceptibility [22]. This is exceptionally notable in low socioeconomic areas in which overcrowding and poverty have been associated statistically with increased substance abuse. In addition, repeated exposure to successful high-status role models who use substances, whether these role models are figures in the media, peers or older siblings, is likely to influence children and adolescents. Similarly, the perception that smoking, drinking or drug use is standard practice among peers also serves to promote substance abuse.

When examining drug addiction through this triple-perspective, we are forced as a global society to re-evaluate the criminalization of drug use and addiction throughout world. In general, social drug policies have been conservative and unyielding. Most often, addicts are left to feed their addiction through illegal means of acquiring drugs. As a result of conservative influence in national politics, a "tough on drugs" philosophy that stresses zero tolerance, law enforcement, and abstinence has been adopted. This philosophy neglects the need for medical and psychological treatment of substance addiction.

Columbia University's National Center on Addiction and Substance Abuse report over 75% of state penitentiary inmates require drug abuse treatment, but the disconcerting fact is that under 20% of those individuals actually are provided with proper treatment programs [23]. If treatment is provided, it is often times extremely short-term and non-intensive, and even less frequently offered to jail inmates. In addition, the Bureau of Justice Statistics stated that only 1 in 10 state prison inmates were provided drug abuse treatment in 1997, down from the 1 in 4 inmates offered treatment in 1991. This is astonishingly low, considering the correctional institution holds more substance abusers than any other national institution. Also commonly noted are the incredibly high comorbidity rates between mental illness and drug addiction within the prison system. It is vital to view substance addiction as a medical condition when dealing with criminal charges, making sure that addicts are provided with treatment for the root of their affliction rather than simply punishing the active symptoms of addiction.

## 7. Conclusion

Drug use and addiction seem to have been a part of mammalian society since ancient times. Researchers have evidence and reason to believe that the evolution of mammalian brains and psychotropic plants might be related to each other, connected by ancient drug use. Regardless of the possible co-evolution of drugs and mammalian brains, abuse of drugs inevitably causes long-term disadvantages. Drug addiction could be extremely detrimental for any individual, not only because of the various health problems involved, but also due to the fact that it abolishes negative emotions, such as pain, which in turn shuts off basic defense mechanisms against potential threats. While the origins for drug addiction may indeed be genetically founded, abuse is most likely caused by a combination of both external and internal stimuli. Although a person may be pre-disposed to addiction, environmental and emotional stimuli may act as a catalyst towards the state of actual substance addiction. It is suggested that the motivation towards drug abuse comes from reward systems within the mammalian brain causing an initial "like" for a substance and leading to the insatiable "want" that correlates with abuse. Although there has been a distinction made between a possibility of a reward-based abuse and a salience-based abuse, it may be possible to see a combined effort of the two proposed systems working towards eventual drug addiction.

More research spanning the evolutionary history of mammalian brains might give us a greater awareness of the physiological wiring of the mammalian brain. For example, is there a combined influence of the salience and reward systems? Are these systems in fact hard-wired, indicating a hard-wired and possibly genetic underlying origin of liability to drug abuse? What is the true reason all humans are vulnerable to drug abuse? Are these tendencies towards drug abuse preventable or simply treatable? These and other questions may, in turn, allow us a deeper understanding of how to effectively prevent and treat drug abuse without simply placing a bandage over it by relieving the superficial symptoms accompanying it. Essentially, we must investigate what may universally cause this internal affliction before we can move on to examine external environmental stimuli that may be associated with individual cases.
